# CT Perfusion Imaging Guides Clinical Decision-Making in a Case of Thalamic Stroke: A Case Report

**DOI:** 10.7759/cureus.44846

**Published:** 2023-09-07

**Authors:** Alisha Qaiser, Daniela Lozano, Nicholas Liquigli, Kasim Qureshi, Muhammad Farooq

**Affiliations:** 1 Neurology, Trinity Health Saint Mary's - Grand Rapids, Grand Rapids, USA; 2 Vascular Neurology, Trinity Health Saint Mary's - Grand Rapids, Grand Rapids, USA

**Keywords:** artery of percheron infract, case report, cerebrovascular disease, ct brain perfusion, neuroimaging, vascular neurology, stroke

## Abstract

This case highlights a patient presenting with a stroke code in the emergency department with decreased consciousness. The patient was later found to have bilateral thalamic strokes due to ischemia of the artery of Percheron. Initial head computed tomography (CT) and CT angiogram (CTA) of the head and neck showed no abnormalities. CT perfusion (CTP) showed a perfusion deficit of 169 mL with a T-max greater than 4 s and 4 mL with a T-max greater than 6 s in the posterior circulation. The patient received IV alteplase. This case report emphasizes the importance of perfusion neuroimaging in the evaluation of acute ischemic stroke.

## Introduction

Computed tomography (CT) perfusion neuroimaging has become a vital component in the initial evaluation of acute stroke because of its ability to identify areas of reduced perfusion and salvageable tissue. In select situations, current guidelines recommend the use of perfusion imaging to help identify patients with a favorable profile for mechanical thrombectomy. The applicability of perfusion findings in other acute stroke scenarios is less established. It is primarily used for thrombectomy selection but can be applied in other settings that include, but are not limited to stroke, anoxic brain injury, and other cerebrovascular disorders such as subarachnoid hemorrhage [1]. CT perfusion (CTP) assesses the circulation of blood flow within different areas of the brain and detects areas of poor blood supply which can help guide further management.

The medial, lateral, and posterior thalamic regions are primarily supplied by the branches of the posterior cerebral and posterior communicating arteries [2]. The branches from the posterior cerebral artery (P1 and P2 segments) include the posterior thalamosubthalamic, thalamogeniculate, and posterior choroidal arteries. Branches of the posterior communicating arteries include the anterior thalamosubthalamic paramedian arteries [2]. There are many variations in the thalamic blood supply, one of the most rare is the artery of Percheron (AOP). It is an anatomical variant that arises from the proximal branch of the posterior cerebral artery and supplies both sides of the subthalamus, thalamus, and rostral midbrain [2]. The estimated prevalence of AOP in the general population is 4-12% and accounts for 4-18% of all thalamic ischemic strokes [3-9]. The thalamus is a crucial area in the brain that is responsible for several functions, such as sensory processing, memory, and motor control. AOP infarct typically presents as confusion, drowsiness, double vision, and difficulty with speech and movement [9]. The decreased arousal is typically caused by infarction involving the paramedian nucleus that contains the intralaminar nuclei, especially when affected bilaterally [10].

It is crucial for the treating physician to be aware of this rare but debilitating condition so that prompt diagnosis and management can be made to improve the outcomes for the patient. We report a case of CTP imaging assisting in clinical decision-making in a case of bi-thalamic stroke secondary to AOP presenting as altered mental status. This case report was previously presented at the 2023 American Academy of Neurology annual scientific meeting.

## Case presentation

**Figure 1:**
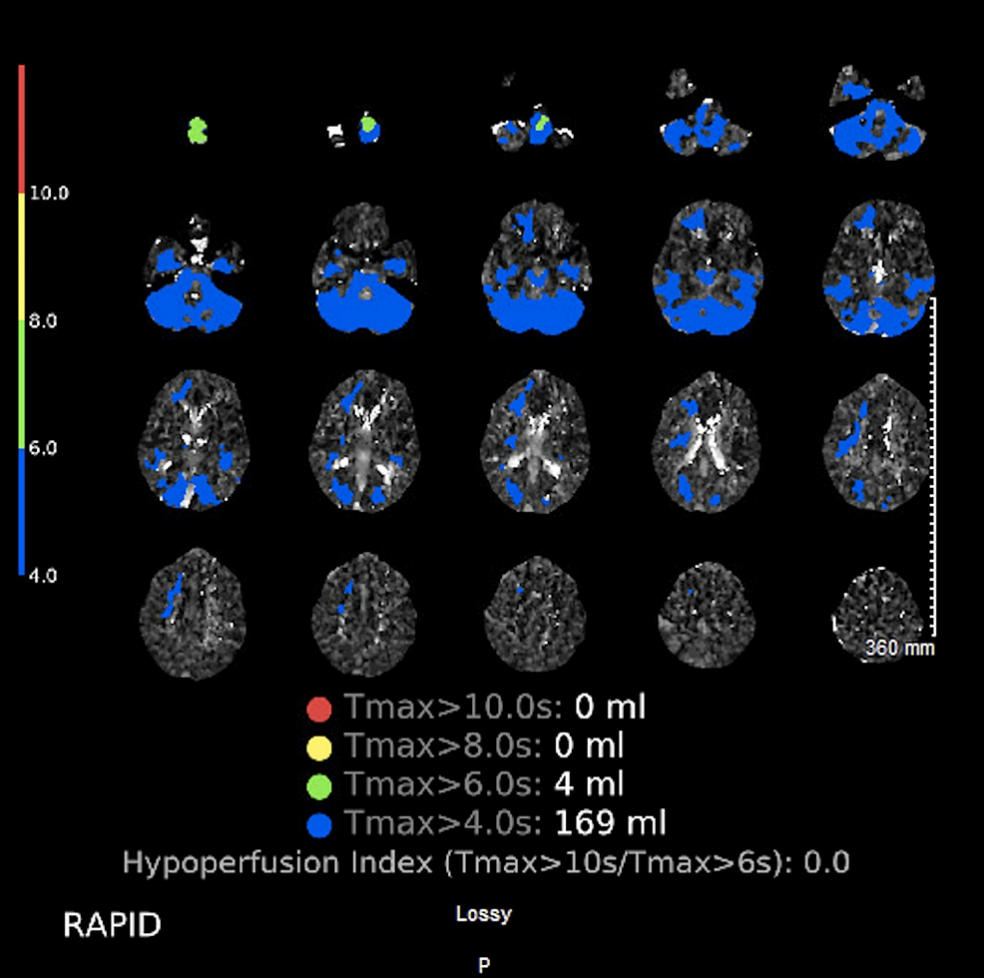
CT perfusion brain T-max values

A 77-year-old female, with hypertension and obesity, presented with altered mental status to the emergency room as a pre-arrival stroke alert after being found unarousable by her partner in bed. Her last known well time was two hours prior to the presentation. On arrival, she had a blood pressure of 127/92 mm Hg and a blood glucose of 98. Her National Institute of Health Stroke Scale was 16. On neurological examination, the patient did not respond to loud auditory stimuli. Her pupils were 2 mm bilaterally and non-reactive to light. Her oculocephalic reflex was absent. She grimaced to sternal rub and could briefly follow the request to give a thumbs up bilaterally but did not open her eyes and would quickly fall back asleep with audible snoring. She produced no speech and withdrew all extremities from painful stimulation. Reflexes were 2+ and symmetric. Her Glasgow coma scale was 9. Initial head CT without contrast and CTA of the head and neck without and with contrast showed no abnormalities. CTP showed a T-max greater than 4 s volume of 169 mL and greater than 6 s volume of 4 mL in the bilateral posterior circulation distribution. Cerebral blood flow was 0 mL in all thresholds from less than 20-38% (Figure 1). The T-max reflects the transit time of the intravascular contrast dispersing throughout the brain parenchyma. The frequently used T-max parameter is greater than 6 s, which provides an estimate of final infarction in patients without reperfusion [11].

**Figure 2:**
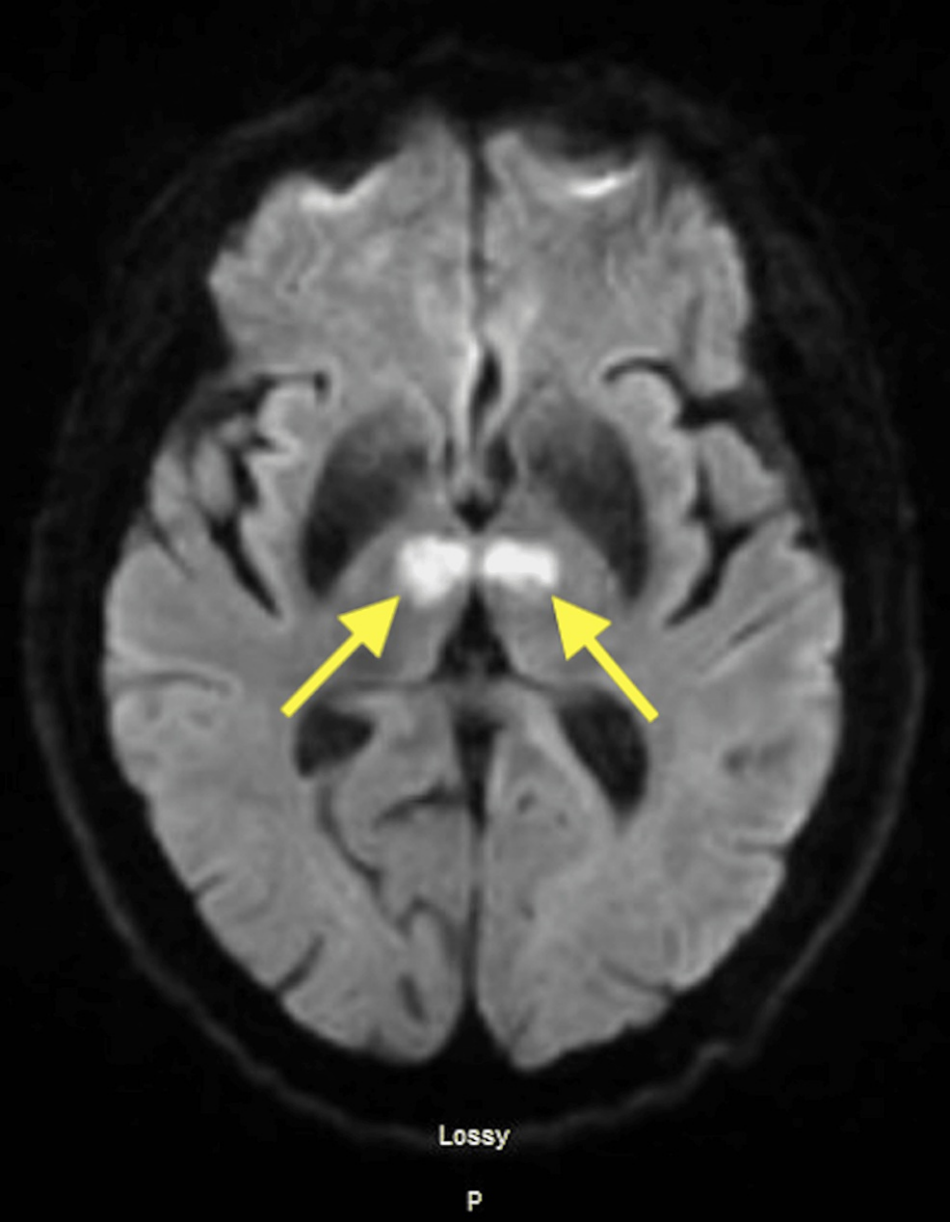
MRI of the brain without contrast demonstrating an area of restricted diffusion on DWI sequence within bilateral thalami reflecting artery of Percheron infarct Restricted area of diffusion seen on DWI sequence (arrows) MRI: magnetic resonance imaging. DWI: diffusion-weighted imaging

**Figure 3:**
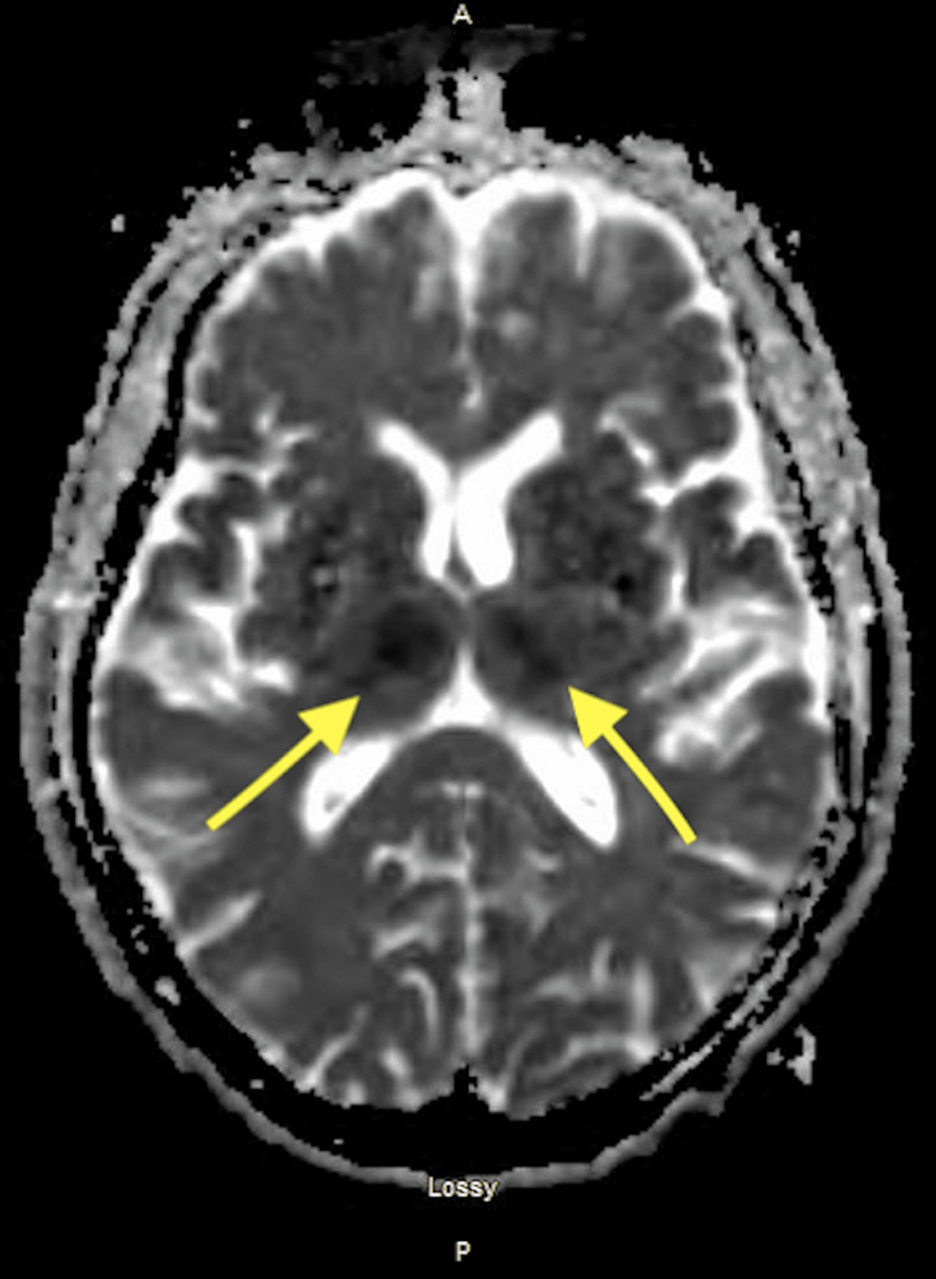
MRI of the brain without contrast demonstrating a decreased signal within bilateral thalami on ADC sequence reflecting the artery of Percheron infarct Decreased signal seen within bilateral thalami (arrows) ADC: apparent diffusion coefficient

Given the patient’s significantly altered mental status, normal blood glucose level of 98, and lack of response to naloxone along with CTP findings, bilateral thalamic stroke was considered. She was administered intravenous alteplase after a discussion of risks and benefits with her husband and was subsequently admitted. MRI of the brain showed diffusion restriction in bilateral thalami (Figures 2-3). Within 24 hours of being given alteplase, her mental status improved, and her blood pressure was maintained within the goal of <180/105 mm Hg. She was somnolent but arousable to auditory and tactile stimulation. She was oriented to her first name only and was noted to have expressive aphasia and dysarthria. Despite being intermittently drowsy, she was able to follow simple motor commands. Blood work revealed an LDL of 104 mg/dL and an A1c of 5.1%. No embolic source was identified. Holter monitor was negative for atrial fibrillation/flutter. She was placed on a long-term loop recorder. One month following discharge she continued to have persistent aphasia and impaired cognition and required 24-hour care. At six months, she had improved in her activities of daily living, mild cognitive deficits, and mild receptive/expressive aphasia.

## Discussion

CTP imaging is achieved by obtaining scans over a minute following IV-iodinated contrast administration [12]. It is able to assess perfusion within the brain tissue as the iodinated contrast enters the cerebral arteries and washes out through the venous system. It is most commonly used for stroke, but aids in characterizing brain tumors, vascular disorders such as aneurysms and arteriovenous malformations, and seizures [13-14]. In cases of refractory epilepsy, CTP can help with preoperative planning as it can help identify areas with abnormal perfusion patterns [15].

According to current AHA/ASA guidelines for early management for patients with acute ischemic stroke, CTA with CTP or MRA with MR perfusion is useful for selecting candidates for mechanical thrombectomy between six and 24 hours after the last known well [3,7]. Our case considers an unconventional application of perfusion neuroimaging beyond current eligibility for mechanical thrombectomy in identifying cerebral tissue at risk. While it is important to be proficient in interpreting perfusion neuroimaging, its relation to the patient’s clinical presentation and knowledge of neuroanatomy is critical in recognizing uncommon presentations of stroke such as altered mental status.

Although helpful, a limitation of CTP imaging is that it must be used with caution in patients with chronic kidney disease as the contrast can be toxic to the kidneys. Further, it may not be as accurate as other imaging modalities, such as MRI in detecting small infarcts. In this case, appropriately interpreting CTP T-max volumes provided valuable information in influencing decision-making for giving IV thrombolytic therapy in a patient with significantly depressed mentation and otherwise nonfocal neurologic exam.

## Conclusions

CTP can be useful in certain circumstances when the clinical diagnosis is unclear on initial presentation. This could greatly impact acute management and long-term outcomes. This case suggests possibly utilizing CTP for patients with altered mental status of unclear etiology, which may help identify near-miss stroke patients.
